# A Thymine Dimer Stalls a High-Fidelity DNA Polymerase by Providing No Template Information in the Same Manner as an Abasic Site

**DOI:** 10.1021/acs.biochem.5c00781

**Published:** 2026-06-22

**Authors:** Amanda R. Walsh, Hye Rhee Kim, Lorena S. Beese, Eugene Y. Wu

**Affiliations:** † Department of Biology, 6888 University of Richmond, Richmond, Virginia 23173, United States; ‡ Department of Biochemistry, Duke University Medical Center, Durham, North Carolina 27710, United States

## Abstract

Cyclobutane pyrimidine dimers are photolesions that form when UV-B irradiation causes adjacent thymine or cytosine bases to covalently bond and can arrest DNA synthesis by replicative polymerases. To gain insight into the effects of cis-syn cyclobutane thymine dimers (T=T) on DNA replication in a model polymerase, we conducted solution and crystallographic studies of the Bacillus DNA polymerase I large fragment (BF) in complex with a T=T-containing template and an incoming dNTP. A thymine dimer lesion blocked nucleotide addition by BF in solution. The crystal structure of the BF-T=T-dATP complex indicated that the thymine dimer is too large to enter the template insertion site, preventing the lesion from providing any information to copy. We compare the T=T ternary complex with a BF ternary complex with an abasic site analogue and show that both lesions are noninstructional and induce nearly identical conformations in the polymerase and the substrate dATP. The incoming dATP inserts too far into the active site in the absence of a template base and distorts into a structure that is not conducive for catalysis with the primer 3′-hydroxyl. Together, our results show that thymine dimers and abasic sites can stall DNA synthesis by providing an absence of information through similar mechanisms.

## Graphical abstract

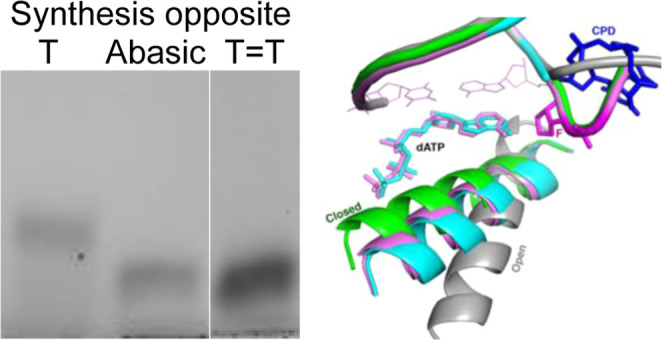

## Introduction

The fidelity of DNA replication is essential to genomic stability. DNA polymerases accomplish the task of replication accurately and efficiently through rapid conformational changes that enable the enzyme to sample and reject incorrect nucleotide substrates before phosphoryl transfer.^1^ High-fidelity DNA polymerases generally operate using a complex-induced-fit mechanism. In the binary (polymerase-DNA) complex of DNA polymerase I from *Geobacillus stearothermophilus*, large fragment (Bacillus Fragment, or BF), the acceptor template base is flipped out of the DNA base stack, occupying a position known as the preinsertion site.^2^ When a complementary nucleotide substrate binds the binary complex at the insertion site, the O-helix of the mobile finger domain of the polymerase transitions from an open to a partially closed (or ajar) conformation and then a closed conformation if the nucleotides form a Watson–Crick base pair, enclosing the dNTP opposite the acceptor template base in a ternary complex.^3–5^ The proper alignment of the dNTP for inline attack by the 3′-hydroxyl of the growing DNA strand leads to phosphodiester bond formation. Replicative DNA polymerases insert nucleotides with remarkable specificity and speed. However, damaged DNA bases can pose a threat to fast and accurate replication by impairing the mechanism of nucleotide selection and insertion.

Two common and harmful types of DNA damage are the cyclobutane pyrimidine dimer (CPD) and the abasic site. CPDs are premutagenic DNA lesions which form in skin cells after exposure to solar UV radiation. When UV-B (290–320 nm) activates the C5=C6 double bond of a pyrimidine base, the pyrimidine can form two covalent bonds with a neighboring pyrimidine resulting in a CPD, the most prevalent of which is a cis-syn thymine dimer.^6^ The formation of these aberrant photoproducts is common; approximately, 20 CPD lesions are formed per 10^6^ nucleotides when human skin is irradiated by solar-simulated UV radiation.^7^ Similarly, ∼13 abasic sites exist per 10^6^ nucleotides in replicating eukaryotic cells and 30,000 abasic sites exist per cell despite active repair of these lesions. Abasic sites are generated by a variety of different chemical pathways, including spontaneous depurination/depyrimidation, active depurination/depyrimidation by DNA glycosylases during DNA repair, and oxidative damage.^8^ Cells have developed various types of DNA damage tolerance mechanisms to repair these lesions, including nucleotide excision repair and photoreaction repair.^9^ Despite continual repair of these two common lesions, some CPDs and abasic sites can remain and pose a threat to replication by stalling replicative polymerases.^10,11^ For some polymerases, both thymine dimers and abasic sites act as noninstructive lesions, stalling DNA synthesis by providing no template for replicative DNA polymerases with constrained active sites.^12,13^ Because of the drastic change in sterics, replicative DNA polymerases with high fidelity may not accommodate large adducts into their tight active sites, which typically accommodate only one template base at a time.

When replicative polymerases stall at a damaged DNA site, specialized polymerases, such as those from the Y-family, are often recruited to the primer terminus to perform translesion synthesis.^14,15^ These polymerases have wider active sites that are better suited for accommodating and synthesizing past bulky photolesions.^16^ Some of the Y-family polymerases, such as bacterial DNA polymerase V and eukaryotic DNA polymerase eta (Pol η), can copy past CPDs with accuracy.^15,17,18^ However, a wider, less discriminatory active site makes several of the Y-family polymerases generally more prone to error.^19^ Because of their low fidelity, replication past lesions by certain Y-family polymerases sometimes results in carcinogenic transition mutations.^14^ Thus, tight regulation is essential to ensure that these enzymes are recruited only when absolutely necessary.^14^ When transition mutations occur in key regulatory genes such as the *p53* tumor suppressor gene, basal cell or squamous cell carcinomas can occur. CPD-derived transition mutations in *p53* have been found to occur in 50 percent of all human basal cell carcinomas and are therefore considered the signature mutation of UV-B radiation.^20^

To better understand the structural basis for the stalling of DNA replication by these common lesions, we show that a cis-syn thymine dimer indeed cannot enter the active site of a replicative DNA polymerase and, like abasic sites, provide no template information using BF DNA polymerase as a model polymerase. The crystal structure of BF complexed with a dATP substrate and a thymine dimer or an abasic site shows that the nucleotide inserts too deeply into the active site to properly form a phosphodiester bond with 3′-hydroxyl, explaining why the BF polymerase is extremely slow at replicating past both lesions. In contrast to Y-family polymerases, BF’s constrained and narrow insertion site makes strong use of base stacking to manipulate the positions of the template and substrate.

## Materials and Methods

### Materials

The gene sequence encoding residues 297–876 of DNA polymerase I from *G. stearothermophilus* (Uniprot E1C9K5)^21^ were synthesized and cloned into pET21a immediately behind the NdeI site and its start codon (Genscript, Piscataway, New Jersey). The mutations of aspartic acid 598 to alanine (D598A) and phenylalanine 710 to tyrosine (F710Y) were generated by site-directed mutagenesis (Genscript). The expression plasmids were transformed into BL21­(DE3)­pLysS *Escherichia coli* cells and grown to log phase. Protein expression was induced with isopropyl β-D-1-thiogalactopyranoside when the culture absorbance at 600 nm reached ∼0.6. The culture was shaken for 4 h at 37 °C and then harvested by pelleting in a centrifuge. The bacteria were frozen at −20 °C overnight, thawed, and then lysed with B-PER detergent (Thermo Fisher Scientific, Waltham, Massachusetts), lysozyme (Hampton Research, Aliso Viejo, California), and Pierce Universal Nuclease for Cell Lysis (Thermo Fisher Scientific) according to manufacturer’s instructions. Cell lysates were heated to 65 °C for 10 min to denature some of the cellular proteins and then separated by centrifugation at 15,000*g* for 5 min at 4 °C. Purification of BF protein via ion exchange chromatography and heparin sulfate affinity chromatography was performed as previously described.^3^

Primer and template oligonucleotides were synthesized by TriLink Biotechnologies (San Diego, CA) or Eurofins Genomics (Louisville, KY). Equimolar amounts of complementary primers (5′-[6FAM]-GTCAAGGACAGTG-3′ (complexes 0TT and 0F), 5′-[6FAM]-GTGCATAGCCAGTCTCGGGTGTGATTGCTCTAATCCATCAACGTGTCAAGGACAGTG-3′ (complex I), 5′-CAAGGACAGT-3′ (complex II), and 5′-CGATCACG-3′ (complex III)) and templates (5′-ATCACTGTCCTTGAC-3′ (complex 0), 5′-A**T**=**T** CACTGTCCTTGAC-3′ (complexes 0TT, I, and II), 5′-A**F** CACTGTCCTTGAC-3′ (complex 0F), and 5′-GACG**F**ACGTGATCG-3′ (complex III)) were annealed by heating to 85 °C and then slowly cooling 1° per minute to 25 °C. Typically, 8–10 base pairs are needed to create a stable duplex. **T**=**T** is a cis-syn thymine dimer, **F** is tetrahydrofuran, and complementary sequences are underlined. See Table 1 for each annealed complex.

**Table 1:** Primer–Template Design for Kinetics and Crystallography Experiments^a^

experiment	name	duplex sequence
kinetics	complex 0	5′-[6FAM]-GTCAAGGACAGTG
		3′-CAGTTCCTGTCACTA-5′
	
	complex 0TT	5′-[6FAM]-GTCAAGGACAGTG
		3′-CAGTTCCTGTCAC**T=** **T**A-5′
	
	complex 0F	5′-[6FAM]-GTCAAGGACAGTG
		3′-CAGTTCCTGTCAC**F**A-5′
	
	complex I	5′-[6FAM]GTGC···CGTGTCAAGGACAGTG
		3′-CAGTTCCTGTCAC**T=** **T**A-5′
		
crystallography	complex II	5′-CAAGGACAGT
		3′-CAGTTCCTGTCAC**T=** **T**A-5
	
	complex III	5-CGATCACG
		3-GCTAGTGCA**F**GCAG-5

a **T=** **T** represents the *cis*-syn thymine dimer and **F** represents tetrahydrofuran.

### Solution Kinetics of BF Polymerase

Primer extension by the wild-type BF polymerase was measured in conditions of saturating or nonsaturating concentrations of 2′-deoxynucleoside triphosphates (dNTPs). For nonsaturating dNTP reactions, 2 μM of Complexes 0, 0TT, or 0F were mixed with 50 μM 2′-deoxyadenosine triphosphate (dATP), 50 μM 2′-deoxythymidine triphosphate (dTTP), 2.5 mM magnesium sulfate (MgSO_4_), and last 10 units of BF DNA polymerase (*Bst* DNA Polymerase, Large Fragment, New England Biolabs, Ipswich, MA). A and T are added opposite the 5′-overhang by the polymerase. The reactions were quenched by mixing with an equal volume of TBE-Urea sample buffer (89 mM Tris and boric acid, 2 mM ethylene diamine tetraacetic acid, pH 8.0, 12% Ficoll, 0.01% Bromophenol Blue, and 7 M Urea) at various time points and loaded on a 15% Mini-PROTEAN TBE-Urea gel (Bio-Rad, Hercules, CA). Gels were run for 150 V for ∼60 min, and fluorescent primers were visualized under UV-irradiation in a ChemiDoc Imaging System (Bio-Rad).

To determine if a small amount of primer extension opposite a thymine dimer is occurring at a slow rate that is difficult to detect using gel electrophoresis, a similar quench-flow assay was conducted by mixing a solution containing enzyme (0.1 μM) and Complex I DNA (2 μM) with equal volumes of the four 2′-deoxynucleoside triphosphates (dNTPs) (5–500 μM) and quenching the reaction at time points ranging from 6 to 6000 s with four reaction volumes of a 95% formamide and 20 mM ethylenediaminetetraacetic acid solution (final concentrations). All reagents were diluted to their final concentrations in reaction buffer (50 mM Tris–HCl pH 7.5, 10 mM MgCl_2_, and 50 mM NaCl), and reactions were executed manually on the benchtop at room temperature a single time. The primer lengths and amounts were measured by capillary electrophoresis with fluorescence detection by an ABI3100 Genetic Analyzer at Duke University DNA Analysis Facility, Durham, NC, and the areas under each peak was quantitated using a PeakScanner (Applied Biosystems) to determine the fraction of primers with nucleotide incorporation.

### Crystallization, Data Collection, and Structure Determination

A BF-**T=T**-dATP thymine dimer ternary complex was obtained by incubating 0.1 mM BF D598A with 0.25 mM Complex II DNA (3́-CA overhang prevents BF binding to the wrong end of the DNA), 10 mM manganese sulfate (MnSO_4_), and 1 mM 2′,3′-dideoxyguanosine triphosphate (ddGTP) for 30 min prior to adding 5 mM dATP. Crystals were grown by hanging drop vapor diffusion from 46% (v/v) saturated ammonium sulfate, 10 mM MnSO_4_, 100 mM 2-(*N*-morpholino)­ethanesulfonic acid (MES), and 5% 2-methyl-1,3-propanediol (MPD). Large, hexagonal crystals that were approximately 300 μm in size began to appear after 2 days, and several smaller crystals (approximately 100 μm) continued to grow in the same drop over the next 20 days. These smaller crystals were screened with several cryoprotectants and then cryoprotected in 46% (v/v) ammonium sulfate, 100 mM MES, 10 mM MnSO_4_, 5 mM dATP, 5% MPD, and 25% (v/v) ethylene glycol. Crystals were then plunged in liquid nitrogen. Diffraction data sets for the ternary crystal were collected at beamline APS 22-BM of the Advanced Photon Source, Argonne National Laboratory (Argonne, IL) (Table 2). Data sets were indexed, integrated, and scaled with XDS^22^ and phased by molecular replacement using PDB 3HPO,^3^ and Phaser.^23^ Coot and REFMAC5 were used to edit the model and perform model refinement.^24,25^ The crystal structure was evaluated by MolProbity.^26^

**Table 2:** Crystallography Statistics

data collection	*T*= T ternary complex	abasic site ternary complex
space group	*P*3_1_21	*P*2_1_2_1_2_1_
cell dimensions *a*,*b*,*c* (Å), α, β, γ (°)	92.14, 92.14, 189.69, 90.0, 90.0, 120.0	93.875, 109.068, 150.096 90.0, 90.0, 90.0
resolution (Å)	79.8–2.25 (2.31–2.25)^a^	46–1.80 (1.89–1.80)^a^
no. of reflections	44,763 (3028)^a^	138,448 (21,082)^a^
*I*/σ(*I*)	21.6 (1.4)^a^	12.27 (2.79)^a^
percent completeness	99.3 (92.4)^a^	96.9 (99.7)^a^
redundancy	6.9 (4.6)^a^	4.6 (4.5)^a^
*R*_sym_	0.063 (1.0)^a^	0.078 (0.595)^a^
nonhydrogen atoms in the asymmetric unit	5305	11,204
		
refinement		
*R*_work_ */R*_free_	0.2241/0.2844	0.2003/0.2423
average *B* factors		
protein	57.4	33.0
DNA	85.2	35.4
waters	45.4	34.3
ligands/ions	71.3	36.2
root-mean-square-deviation		
bond lengths (Å)	0.0087	0.018
bond angles (°)	1.920	1.347
Ramachandran outliers	2 (0.35%)	2 (0.17%)
MolProbity score	2.35 (66th percentile)	3.66 (98th percentile)

a Values in parentheses are for the highest resolution shell of data.

A BF-**F**-dATP (abasic site) ternary complex was obtained by incubating 0.1 mM BF D598A/F710Y with 0.25 mM Complex III DNA, 10 mM magnesium sulfate (MgSO_4_), and 1 mM 2′,3′-dideoxythymidine triphosphate (ddTTP) for 30 min prior to adding 4 mM dATP. Crystals were grown by hanging drop vapor diffusion from 50% (v/v) saturated ammonium sulfate, 20 mM MgSO_4_, 100 mM MES, and 1% MPD. The crystals were soaked in 54% sat. (NH_4_)_2_SO_4_, 100 mM MES pH 5.8, 20 mM MgSO_4_, and 4 mM dATP and then plunged in liquid nitrogen. The diffraction data set for the ternary crystal was collected at beamline 22-ID of the Advanced Photon Source, Argonne National Laboratory (Table 2). Data sets were indexed, integrated, and scaled with XDS^22^ and phased by molecular replacement using PDB 2HVI, which also used the D598A/F710Y BF double mutant.^27^ Coot was used to edit the model, and PHENIX and REFMAC5 were used perform model refinement.

## Results and Discussion

CPDs have long been known to slow or stop DNA replication forks in prokaryotic cells, eukaryotic cells, and even in viruses.^28–30^ In human cells, these UV-induced DNA lesions can lead to mutagenic bypass replication, stalled forks, and carcinogenesis.^31,32^ To examine the effects of encountering a UV-damaged DNA (Complex 0TT) on the polymerase’s rate of catalysis, we measured the solution kinetics of dATP incorporation opposite a thymine dimer in wild-type BF. Extension of DNA opposite the thymine dimer was undetectable at dNTP concentrations less than 100 μM^33–35^ even after a reaction time of 6000 s (Figure 1A). In contrast, BF polymerase efficiently extended past an undamaged template (Complex 0). These data indicate that at physiological dNTP concentrations of 5–37 μM,^33^ BF, like other bacterial A-family polymerases,^34,35^ is incapable of inserting a nucleotide substrate adjacent to a thymine dimer, thereby blocking replication. However, when the dNTP concentration was increased to 100 μM in the presence of excess polymerase, a small fraction of DNA containing a thymine dimer (complex I) was extended by one nucleotide at 0.1 μM BF polymerase after 1800 and 6000 s (Figure 1). These results confirm that, like other replicative polymerases, BF is incapable of translesion synthesis past a thymine dimer.

**Figure 1:**
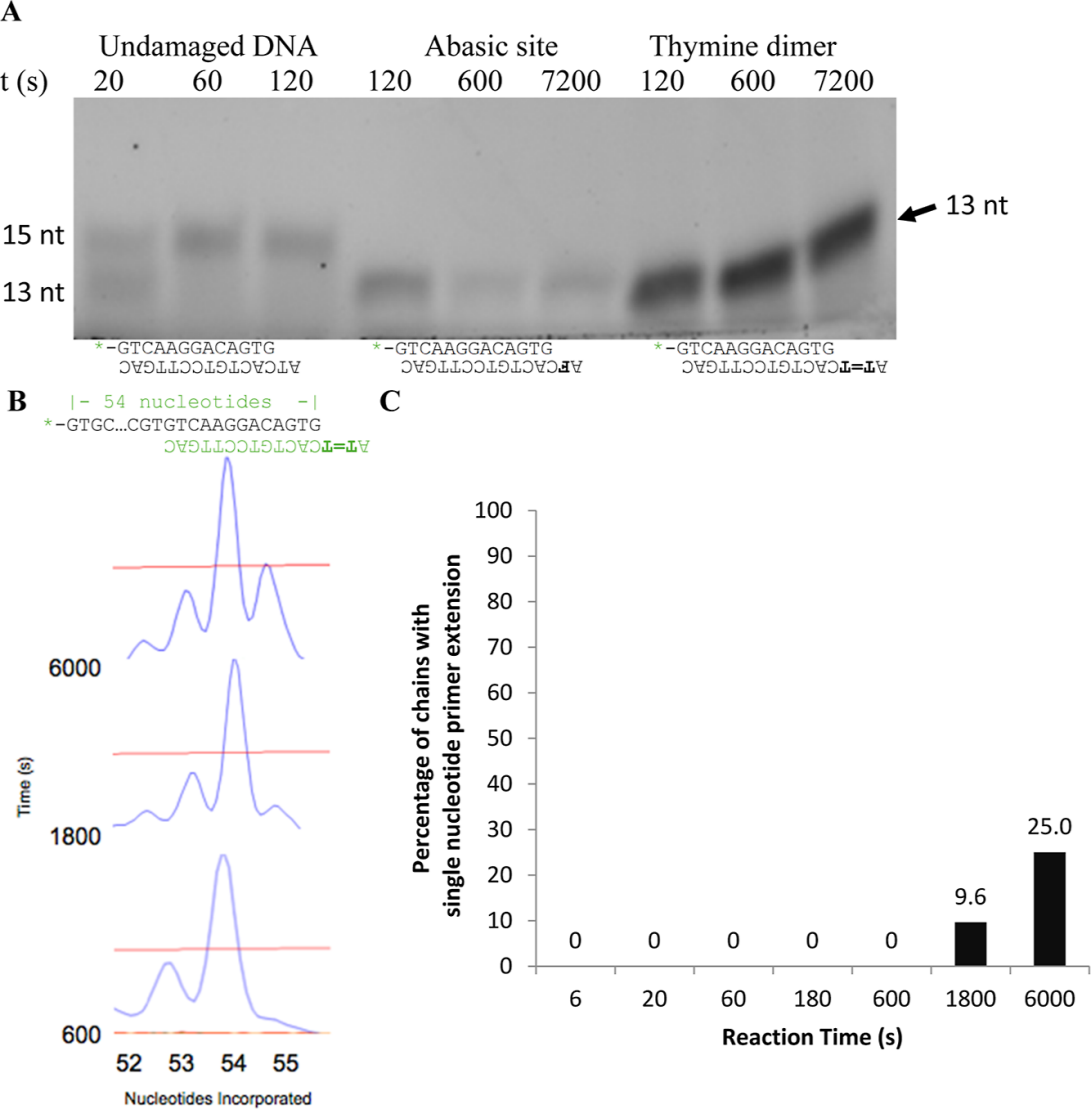
Both an abasic site and a thymine dimer stall nucleotide incorporation by BF polymerase. (A) Denaturing polyacrylamide gel electrophoresis of fluorescent primer extension by BF polymerase at less than saturating concentrations in the presence of 50 μM dATP and dTTP. The 5′-overhang contains a 3′-dTA for undamaged DNA samples, an abasic site analog (**F**, tetrahydrofuran) followed by A for abasic site samples, and a cis-syn thymine dimer (**T=** **T**), followed by an A for thymine dimer samples. (B) At saturating polymerase conditions, capillary electropherograms show the emergence of a new peak (∼55 nucleotides) beginning at 1800 s, indicating small amounts of primer extension opposite the CPD. (C) Integration of electrophoretic peaks indicate no detectable primer extension occurs prior to 1800 s.

In order to better understand the mechanism by which CPDs prevent nucleotide incorporation in the *Bacillus* polymerase, we crystallized BF D598A in complex with a template containing a cis-syn thymine dimer (complex II) and an incoming dATP substrate (Figure 2A). Wild-type BF tends to crystallize in the binary complex^2^ where the finger domain is locked in the open conformation due to crystal packing. This crystal form is stabilized by a salt bridge formed by aspartic acid 598, so a mutation to alanine (BF D598A) was used in order to crystallize the protein in the ternary complex.^27^ Because we were interested in the conformation of the dNTP substrate, we chose to trap the ternary complex by incorporating a chain-terminating 2′,3′-dideoxynucleotide (ddG) into the primer strand of complex II to prevent nucleotide incorporation instead of using a modified, nonhydrolyzable dNTP analogue. Although the solution nucleotide incorporation data do not tell us which dNTP was selected for insertion opposite the 3′ thymine of the thymine dimer, previous studies of lesion bypass indicated that adenosines are preferred by polymerases opposite thymine dimers.^36–38^ The thymine dimer could base pair with dATP according to base-pairing rules. Under these conditions, there could be an untemplated insertion of dATP opposite the 3′ thymine of the thymine dimer in accordance with the “A-rule”, which predicts that adenine nucleotides are preferentially added opposite DNA photolesions and abasic sites.^12,37^ We therefore added dATP opposite the CPD to capture the enzyme in the ternary complex. A single crystal diffracted to 2.25 Å resolution and the data were phased by molecular replacement (*R*_work_ = 0.2241; Table 2).

**Figure 2:**
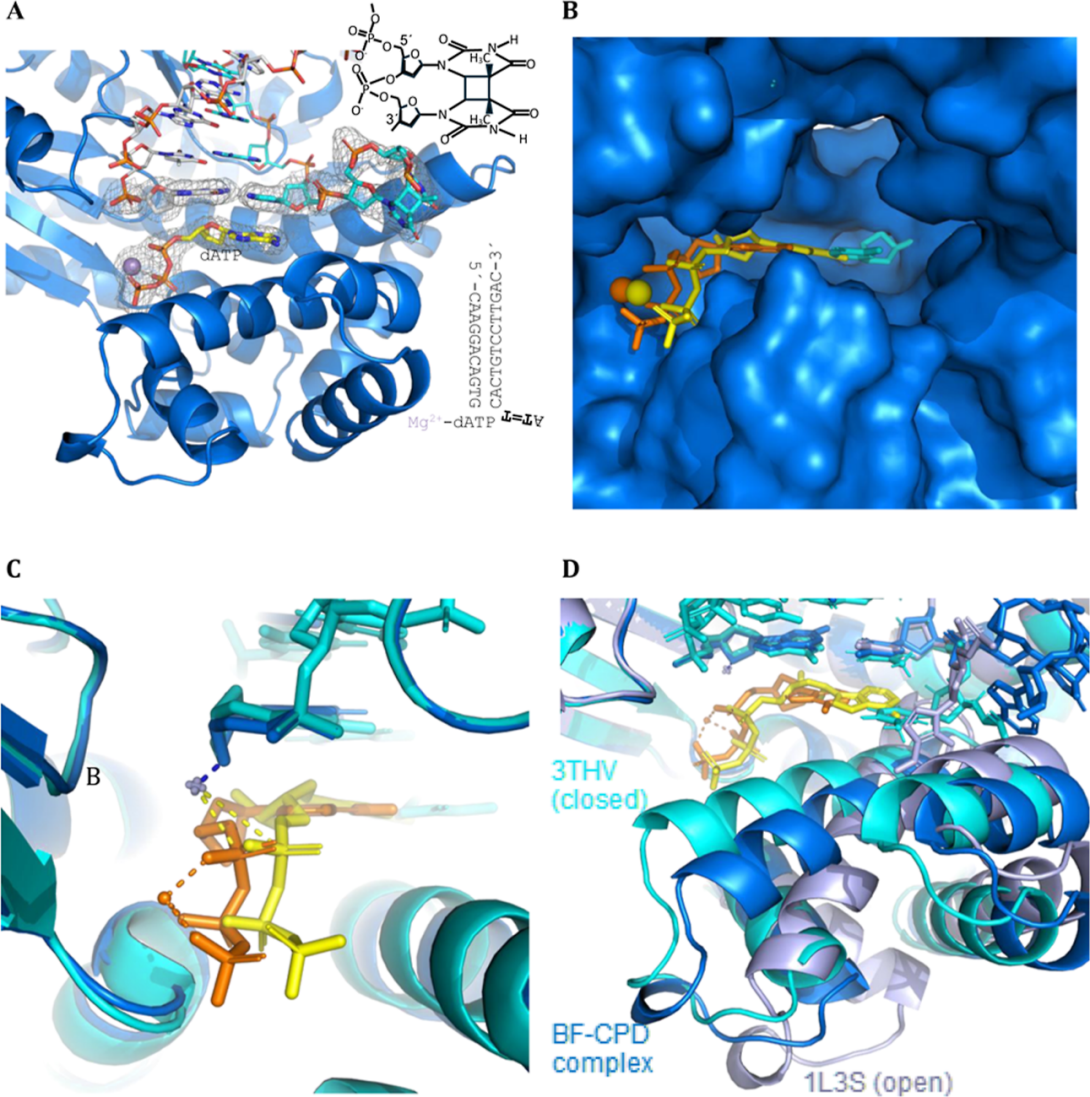
Crystal structure of the BF D598A-**T=T**-dATP ternary complex. (A) The cis-syn thymine dimer (top right inset) ternary complex is shown with BF in blue ribbons, the 2F_O_-F_C_ map at 0.6 σ as a gray mesh, and carbon atoms in the **T=T** template strand in cyan, primer strand in gray, and the dATP substrate in yellow. DNA duplex sequence is shown in the bottom right inset. Phosphorus atoms, orange; oxygen, red; nitrogen, blue; manganese, purple. (B) The solvent-exposed surface of the BF polymerase and bound **T=T**-DNA leaves a gap of approximately 4 Å for the template base, shown with the dATP-Mn^2+^ substrate (yellow) and a superimposed cognate dT (cyan)-ddATP-Mg^2+^ (orange) base pair from PDB 3THV for comparison. (C) A comparison of incoming dNTPs from behind shows ddATP-Mg^2+^ (orange) opposite template dT (PDB 3THV) positioned below a modeled 3′–OH (lavender sphere), while dATP inserted in the active site of the BF D598A-**T=T** = dATP ternary complex (yellow) is not positioned properly for catalysis. (D) A comparison of a BF-DNA open binary complex (lavender; PDB 1L3S), BF-DNA-ddATP closed, ternary complex (cyan; PDB 3THV), and BF D598A-**T=T**-dATP ternary complex (blue) in a position intermediate between the open and closed conformations.

We observed strong electron density for the double-stranded DNA, but no density was observed for the template strand in the insertion site where the templating nucleotide meets the incoming nucleotide. Instead, we observed doughnut-shaped electron density outside the polymerase active site but attached to the template corresponding to the thymine dimer (Figure 2A). Fitting of a cis-syn thymine dimer and the 5′-adenosine into the density revealed that the thymine dimer in the template is rotated out of the active site in the gap where the template strand is fed into the polymerase, and the thymine bases failed to stack with the rest of the DNA template. The thymine dimer appears to float in the template channel and makes few specific contacts with the protein, with only one hydrogen bond between N3 of the 5′ thymine of the dimer and the backbone carbonyl of asparagine 782. The cytosine to the 3′ side of the thymine dimer remains in its normal position in the postinsertion site, base-pairing with ddG in the postinsertion site (Figure 2A). While large modifications in the plane of the template base can be accommodated in the BF polymerase insertion site,^39,40^ the final dC/ddG base pair partners with the polymerase O-helix to form a narrow insertion site of ∼4 Å (Figure 2B), which can accommodate a single nucleobase, but not a pair of covalently bonded bases,^39,40^ and creates steric hindrance orthogonal to the template base that prevents the CPD from being pulled into the insertion site. Manual modeling of a CPD in the BF insertion site with the 3′-thymine as the template base suggests that the 5′-thymidine monophosphate of the CPD would clash with residues in the polymerase O-helix (F710, G711) and point the downstream template toward the O1 helix. The exclusion of the thymine dimer from the insertion site presents no base-pairing information to the incoming nucleotide, thereby impairing selection and insertion of a dNTP substrate into the primer strand. These results are consistent with a previous model that the 3′-pyrimidine of a CPD would have difficulty providing base-pairing information in several A family polymerases due to the inability of the covalently bonded dimer to enter the insertion site.^36^

Another important structural feature in this model is the misalignment of the dATP substrate. Upon meeting a thymine template base, the dATP substrate would typically form a Watson–Crick base pair inside the polymerase active site, positioning the α-phosphate directly below the 3-hydroxyl of the primer strand for in-line attack. Because the thymine dimer is unable to move into the insertion site, the incoming dATP is itself positioned into the insertion site, stacking with the cytosine that is 3′ to the dimer on the template strand (Figure 2A). A comparison between the BF ternary complex (PDB code 3THV)^41^ with a normal dT/ddATP pair and the BF-CPD-dATP complex highlights the change in position of the dATP as a result of the absence of a template base (Figure 2B). The deeper insertion of the adenine base into the insertion site leads to the misalignment of the triphosphate moiety relative to the primer terminus (Figure 2B,C). Because the 3′-hydroxyl of the primer strand is missing in the crystal structure, an oxygen atom was manually modeled near the 3′-carbon of the final nucleotide by aligning a crystal structure containing a 3′-hydroxyl (PDB 1L3S).^2^ The distance between a modeled 3′-hydroxy of the primer terminus and the α-phosphorus atom of the incoming increases from 3.8 Å in a closed ternary complex to 5.1 Å in the misaligned BF-CPD-dATP complex (Figure 2C). In addition, the 3′-oxygen of the primer and the triphosphate are not in line for nucleophilic attack (Figure 2C), explaining the inhibition of nucleotide addition opposite the CPD. This misalignment of the dNTP substrate impedes catalysis and therefore inhibits primer extension.

Third, there was a notable change in the position of the mobile O-helix of the fingers domain (Figure 2D). The formation of a Watson–Crick base pair between the incoming nucleotide and the template base induces a large conformational change in the fingers subdomain, specifically the rotation of the O-helix by ∼40–45°.^2,42,43^ Here, BF fails to completely clamp the dATP in the closed conformation due to the deep insertion of the dATP substrate, inducing only a ∼25° rotation (Figure 2D). Such intermediate states have been observed in the BF crystal structures of mismatched nucleotides or ribonucleotides in the active site,^3,41,44^ and partially closed states have been observed in solution in association with mismatches.^4,45^ Molecular dynamics simulations also support the existence of a partially closed state that serves as a checkpoint for proper base pairing.^5,46^ The dATP, with no information to guide it, is unable to induce BF to transition to the closed state and is trapped at the checkpoint. This suggests that the inability of the CPD to properly align and position the incoming dATP impedes full rotation of the O-helix, contributing to the loss of polymerase activity.

We sought to compare the CPD ternary complex with a polymerase encountering an abasic site, a DNA lesion with no base for pairing. Abasic sites (also known as apurinic/apyrimidinic sites) are frequently generated by spontaneous hydrolysis of a nucleotide’s glycosidic bond or during base excision repair.^12^ The resulting natural abasic site is prone to ring opening,^47^ so we substituted tetrahydrofuran as a stable analog of an abasic site. Parallel solution experiments indicated that, like a thymine dimer, the abasic site blocks DNA synthesis by BF polymerase, showing no detectable primer extension even after 7200 s (Figure 1A). We solved the crystal structure of the ternary complex of BF bound to an abasic site opposite a dATP to 1.80 Å resolution (*R*_work_ = 0.200; Table 2). Like in the thymine dimer ternary complex, the dATP inserts deeply into the insertion site of the polymerase, stacking its adenine base with the previous template base (Figure 3A). The substrate adenine takes the place of the missing template base. The polymerase fingers subdomain adopts a partially closed conformation nearly identical to that of the thymine dimer ternary complex (Figure 3B). The abasic site-DNA template backbone, however, is similar to an undamaged DNA template, with the abasic site facing the substrate dATP, whereas the thymine dimer, including its backbone sugar, is entirely excluded from the active site. These structures indicate that CPDs and abasic sites induce similar conformations of BF while providing no base-pairing information.

**Figure 3:**
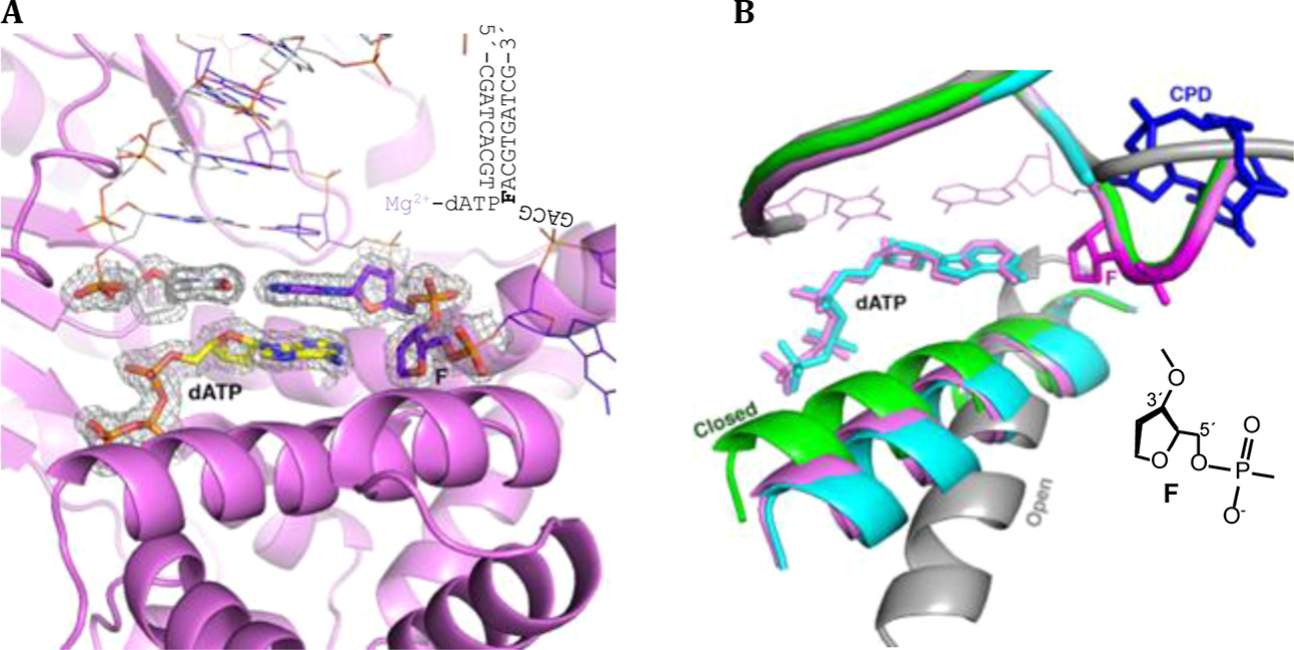
Abasic site analogue forms a nearly identical complex with BF and dATP. (A) The tetrahydrofuran (labeled **F**) ternary complex with dATP is shown at 1.80 Å resolution. Protein is modeled in violet ribbons and the 2F_o_–F_c_ map at 1 σ is shown as a mesh. Carbon atoms of the abasic site template are colored in purple, primer in gray, and incoming dATP substrate in yellow. Phosphorus atoms are colored in orange, oxygen atoms in red, and nitrogen atoms in blue. DNA duplex is shown in the top right inset. (B) The abasic site ternary complex (violet) and the CPD ternary complex (cyan) are superimposed with BF in the open (gray; PDB code 1L3S)^9^ and the closed (green; PDB code 2HVI)^34^ conformations. The DNA backbones are shown as thick tubes, and the postinsertion site base pair is shown as thin lines. The chemical diagram for **F** is shown below the structure.

Our results (Figure 4A) differ from a 3.2 Å crystal structure of ddATP bound opposite the 3′ thymine of a cis-syn thymine dimer in the template strand in a fellow A-family T7 DNA polymerase (PDB code 1SL0; Figure 4B), which showed the enzyme in the open conformation, with insufficient electron density to model the thymine dimer or nearby residues in the enzyme due to disorder.^13^ The ddATP is bound along the O-helix of T7 DNA polymerase but not inserted into the active site nor stacked with the bases in the double helix as in our structure. Our CPD ternary crystal structure shows that the CPD is occluded from entering the polymerase insertion site and serves as a noninstructional lesion, like an abasic site. Several polymerases in the Y-family, including as human Pol η, Pol ι, and Pol κ, have been shown to efficiently bypass *cis*-syn thymine dimers in vitro.^16^ These polymerases are characterized by lower fidelity and processivity, with loose active sites that facilitate nucleotide incorporation opposite damaged DNA. Crystal structures of CPD in the active sites of human Pol η (Figure 4C) and Pol κ show that the path taken by the template in Y-family polymerases is wider than in A-family polymerases, allowing a large lesion like CPD to interact with an incoming dATP at the 3′- and 5′-thymidine of the CPD.^48–50^

**Figure 4:**
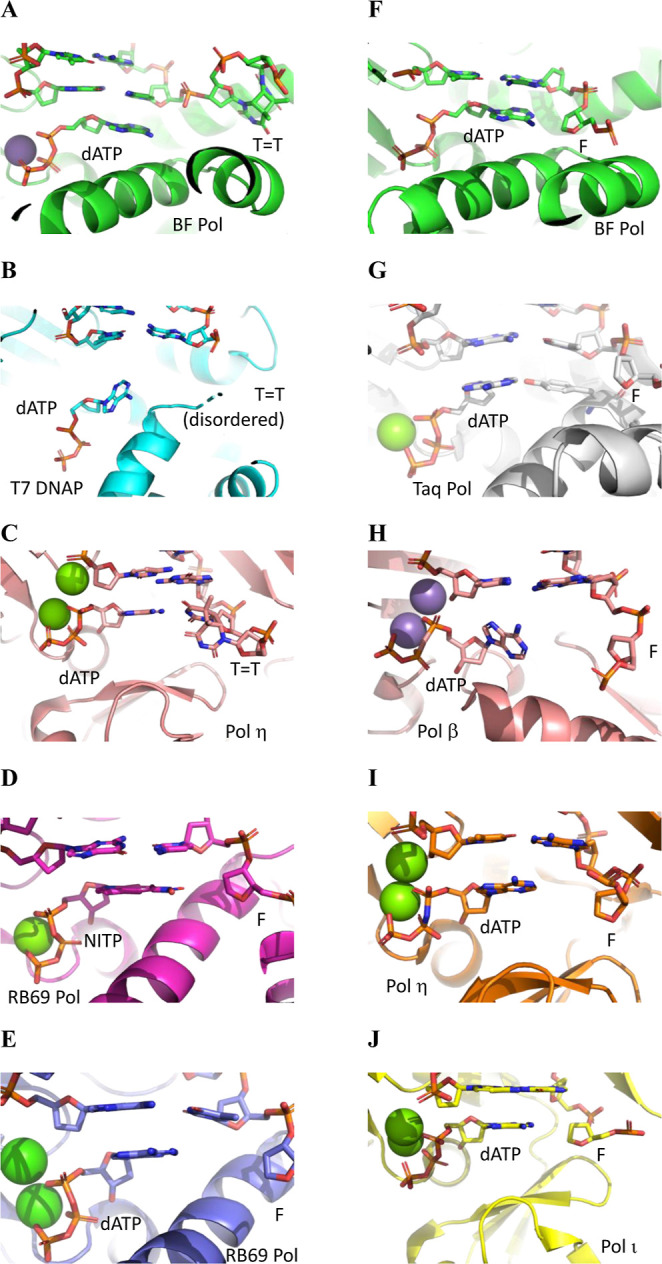
Structures of polymerase ternary complexes containing a noninstructional DNA lesion in the template. All active sites are oriented to show the last base pair in the double-stranded DNA near the top of the figure, the incoming nucleotide at the left, and the DNA lesion at the right. Mg^2+^ and Mn^2+^ divalent cations are shown as green and purple spheres, respectively. (A) BF DNA polymerase bound to a thymine dimer and a dATP from this study; (B) T7 DNA polymerase bound to a thymine dimer (unstructured) and a ddATP (PDB 1SL0); (C) DNA polymerase η bound to a thymine dimer and a dATP (PDB 3MFI); (D) RB69 DNA polymerase bound to an abasic site and NITP (PDB 2OZM) or (E) dATP (PDB 4DTN); (F) BF bound to an abasic site and a dATP from this study; (G) Taq DNA polymerase (Y671 in center) bound to an abasic site and ddATP (PDB 3LWL); (H) DNA polymerase β bound to an abasic site and dATP (PDB 3ISD); (I) DNA polymerase η bound to an abasic site and a dATP (PDB 4RNM); and (J) DNA polymerase ι bound to an abasic site and a dATP (PDB 3G6V).

In addition to the width of the template insertion site, base stacking also plays an important role in the positioning of the incoming dNTP. In BF polymerase, the adenine base of the dATP is dragged into the template insertion site by stacking directly below the template base to the 3′ side (*n*–*1*) of the noninstructional lesion, be it a pyrimidine (Figure 4A) or a purine (Figure 4F). The importance of π stacking is also seen in a crystal structure of RB69 DNA polymerase (B family) bound to a 5-nitro-1-indolyl-2‘-deoxyriboside-5′-triphosphate (NITP) opposite an abasic site template (Figure 4D), where the 5-nitro group stacks neatly with the cytosine in the *n*–*1* template position and the indole ring stacks with the guanine in the *n*–*1* primer position.^51^ Although dNTP stacking with the *n*–*1* template base is not essential in RB69 DNA polymerase (Figure 4E),^52^ incorporation of purine nucleotides was much more efficient than pyrimidines opposite an abasic site, suggesting that stacking with the *n*–*1* primer base does contribute to nucleotide selection for dATP (“the A-rule”)^12^ when no template information is available. The deeper insertion of dATP in the BF active site also contrasts with fellow A family DNA polymerase I from *Thermus aquaticus* (Taq DNA polymerase) bound to a ddATP opposite an abasic site (Figure 4G).^53^ In Taq DNA polymerase, a conserved tyrosine 671 remains stacked against the *n*–*1* template base in the insertion site, preventing the substrate nucleotide from inserting too deeply. The dATP substrate also does not deeply insert into the active sites of other DNA polymerases involved in DNA repair and translesion synthesis (human DNA polymerases β, η, and ι; Figure 4H–J).^53–55^ These structural comparisons to polymerases that are more efficient at translesion synthesis past a noninstructional lesion suggest that a narrow template path, a partially closed kinetic checkpoint, strong base stacking to the *n*–*1* template base, and deep insertion of the dNTP substrate all contribute to stalling a high-fidelity replicative polymerases like BF.

## Conclusions

Our results indicate that UV-induced CPD lesions and common abasic sites can serve as nearly complete blocks to DNA replication. The presence of an unrepaired lesion during replication would cause the stalling of the replication fork, which must be resolved by DNA repair enzymes or error-prone Y-family DNA polymerases such as DNA polymerase IV or DNA polymerase V. These polymerases frequently misinsert G opposite thymine dimers, resulting in transition mutations.^56^ Similar translesion synthesis polymerases in humans, such as PrimPol,^57^ Pol η, Pol ι, and Pol κ, can bypass abasic sites or pyrimidine dimers but have error rates (∼10^–3^) that are much higher^58,59^ than replicative polymerases and may lead to higher frequencies of insertion/deletion^60^ or transition mutations.
